# Physicochemical parameters, sensory profile and concentration of volatile compounds and anthocyanins in beers brewed using potato variety with purple flesh

**DOI:** 10.1038/s41598-023-37284-w

**Published:** 2023-06-21

**Authors:** Alan Gasiński, Joanna Kawa-Rygielska, Agnieszka Kita, Alicja Kucharska

**Affiliations:** 1 Department of Fermentation and Cereals Technology, Faculty of Biotechnology and Food Sciences, Wroclaw University of Environmental and Life Sciences, Chełmońskiego 37, 51-630 Wrocław, Poland; 2 Department of Food Storage and Technology, Faculty of Biotechnology and Food Sciences, Wroclaw University of Environmental and Life Sciences, Chełmońskiego 37, 51-630 Wrocław, Poland; 3 Department of Fruit, Vegetable and Plant Nutraceutical Technology, Faculty of Biotechnology and Food Sciences, Wroclaw University of Environmental and Life Sciences, Chełmońskiego 37, 51-630 Wrocław, Poland

**Keywords:** Nutrition, Agroecology

## Abstract

In the recent years, beer brewers are experimenting with using various substrates, other than traditional barley malt, water, hops, and yeast for beer production, because new adjuncts to the beer brewing can add new sensory and functional properties to this beverage. Novel potatoes with purple or red-colour flesh are a good and cheap starch source and are rich in bioactive components, which could increase the nutritive value of the produced beer. The aim of the study was to determine whether some part of barley malt can be replaced by the potatoes of purple-colour flesh and assessment of properties of such beer. Beer samples showed increased antioxidant activity, higher concentration of anthocyanins and polyphenol compounds, as well as modified composition of volatiles and lower ethanol content. Beer produced with the addition of 30% of purple potatoes showed acceptable organoleptic qualities in the sensory analysis.

## Introduction

Beer is often mentioned as one of the most often consumed beverages around the world, just after water and tea. It is produced by fermenting wort, typically by *Saccharomyces cerevisiae* yeast^1^. Main ingredient of beer wort is barley malt. Beer wort is acquired in the process of mashing, during which the enzymes, which are present in the barley malt hydrolyse the barley starch, releasing fermentable sugars, such as glucose and maltose. However, some other grain additives and other sources of starch can be used during the mashing process. Large commercial breweries often use adjuncts such as corn, rice, sorghum or even pure sugar syrups. Often this substrates are used for purely economic reasons, as the malt is quite expensive starch source, however, some adjuncts are used to change the colour, aroma or taste of the produced beer^2^. Potatoes were used for the beer brewing processes in the past due to the shortages of barley in the war-time^3^, however, unusual additions to the beer today are considered interesting for many of the consumers. Additionally, few researchers have studied addition of the sweet potatoes (*Ipomea batatas*) to the beer and acquired interesting results^4,5^. Conducted studies have shown that sweet potato addition can increase concentration of bioactive phytochemicals and does not make the novel beer unpalatable. However, to this day, no research has been conducted over beers brewed with the addition classical potato, *Solanum tuberosum*. In this work, research has been conducted over novel variety of potato, Provita, which was officially registered in the Polish national list of varieties in 2021. It is the first of the Polish varieties, which is characterised with tubers with purple coloured flesh^6^. Purple and red colours of potato tubers are caused by high concentration of various anthocyanins. These compounds play a crucial role in creating characteristic red or purple hue of many food products, but more importantly, they are characterised with high bioactive potential^7^. The goal of the study was assessment, whether the potato addition to the beer brewing process might not only possibly be used as a method of lowering beer production cost, but whether these novel additives can be used to improve concentration of phytochemicals, which are proven to have beneficial effects on the human organism.

## Materials and methods

### Materials

#### Raw material

Provita potatoes were supplied by Hodowla Ziemniaka Zamarte Company (Zamarte, Poland). Provita potatoes were characterised with 21.34% dry matter content. Pilsner malt (Viking Malt, Strzegom) and Lubelski hops were acquired from Twój Browar company (Wrocław, Poland). Pilsner malt was characterised with extractivity of 81.21%. Lubelski hops were characterised by 4.5% concentration of α-acids.

#### Chemical reagents

Reagents used in this study were diammonium salt of 2,2-azobis (3- ethylbenzothiazoline-6-sulfonate) (ABTS^+·^) (98%, abcr GmbH, Karlsruhe, Germany), 1,1-diphenyl-2-picrylhydrazil (DPPH^•^) radical (95%, Thermofisher, Munich, Germany), 2,4,6-tripyridyl-S-thiazine (TPTZ) (98%) and potassium persulfate (99%) (Sigma Aldrich, Saint Louis, MO, USA), Folin-Ciocalteu reagent, acetic acid (99%), sulfuric acid (99.5%), iron (III) chloride (99%), sodium acetate (99%) (Chempur, Piekary ´Sląskie, Poland), and sodium carbonate (99%) (Stanlab, Lublin, Poland). Standards used in this study for HPLC analysis were dextrins, maltotriose, maltose, glucose, glycerol, peonidine-3-rutinoside, petunidine-3-gluconiside, peonidine-3-gluconiside, chlorogenic acid, neochlorogenic acid, cryptochlorogenic acid. Standards used in this study for GC-FID analysis were acetaldehyde (ethanal), propanal, izobutyraldehyde, butanal, 3-methylbutanal, methanol, 2,3-butanodione, ethyl acetate, 2-butanol, 1-propanol, hexanal, 2-methylpropan-1-ol (isobutanol), isopentyl acetate, 1-butanol, 2-methylbutan-1-ol, 3-methylbutan-1-ol, ethyl hexanoate, 1-pentanol, 2-methylpyrazine, ethyl heptanoate, 2,5-dimethylpyrazine, 1-hexanol, 2,3,5-trimethylpyrazine, ethyl octanoate, furfural, 2,3-diethylpyrazine, ethyl decanoate and phenylethyl alcohol. All standards used for chromatographic analyses were GC-purity grade or HPLC-purity grade. Standards were acquired from the Sigma-Aldrich company (Saint Louis, MO, USA).

#### Biological material

Biological material used in this study was dried *Saccharomyces cerevisiae* yeast Fermentis US-05 from LeSaffre company (Marcq-en-Barœul, France). Yeast was added to the cooled wort (20 °C) in the dose recommended by the producer (0.5 g d.m. per dm^3^ of the wort). Twenty min prior to inoculation, yeast were rehydrated in sterile, distilled water at a temperature of 20 °C, in a ratio of 1 g d.m. of yeast per 20 cm^3^ of water.

### Brewing process

Beer brewing process was simultaneously performed three times, to prepare three different beers with various shares of the purple potatoes in the ‘grain bill’ of the brewed beer. Dry matter content of the potatoes compared to the extractivity of the malt was used to determine, how much of the potatoes must be used instead of the malt. The potatoes substituted malt in the ratio of 21.34:81.21. In the first beer, the control sample (C), 100% of the extract in the ‘grain bill’ came from barley malt. In the second beer (P30), 30% of the assumed extract in the grain bill came from purple potatoes and 70% from the barley malt. In the third beer (P50), 50% of the assumed extract in the ‘grain bill’ came from the purple potatoes and 50% from the barley malt. 3 kg of malt were used to produce beer C; 2.1 kg of malt and 3.4 kg of peeled purple potatoes was used to brew beer P30; 1.5 kg of malt and 5.7 kg of peeled purple potatoes was used to brew beer P50. The purple potatoes, just prior the beer brewing process were peeled and cut into 1 cm thick slices. The slices were boiled in the 90 °C water for 20 min in the separate vessels for beer P30 and beer P50. After the boiling process, water was drained away from the boiled potatoes and potatoes were cooled to 20 °C and then mixed with barley malt (2.1 kg for P30, 1.5 kg for P50). From this point, the brewing was conducted with the same conditions for all the beers, in three different vessels. Barley malt or potatoes/malt mixtures were mixed with 15 kg of water with the temperature of 52 °C. The temperature of the mashes was adjusted to 52 °C and the mashing was conducted with the following time and temperature regime:

- 10 min at 52 °C
- 40 min at 62 °C
- 20 min at 72 °C
- 1 min at 78 °C

Temperature increase between each of the steps was performed at a ratio of 1 °C per 1 min. After the mashing process, mashes were transferred to the filtration vessels and mashes were sparged with additional water with the temperature of 78 °C. The sparging and filtration process was conducted until the extract of wort leaving the filtration vessel was over 2% w/w, measured with handheld refractometer PAL-1 (Atago, Tokyo, Japan). The filtered worts were brought to the boiling point and hop pellets (3 g per dm^3^ of the wort) were added to the worts, which were then boiled for 60 min. After the boiling process, worts were cooled to the temperature of 19 °C with the use of immersion chillers. The chilled worts were filtered and transferred to the disinfected 25 dm^3^ fermenters. Rehydrated yeast was added to the chilled worts to start the process of fermentation and produce beer. Fermenters were kept for 14 days in the refrigerated cabinets with temperature set at 19 °C. After the fermentation process, beer were transferred from the yeast beds on the bottom of the fermenters and bottled with the addition of 6 g of glucose per dm^3^ of the beer to carbonate the beverage. Beer in bottles was stored for 14 days in the temperature of 19 °C, and then used for subsequent analyses.

### Analytical methods

#### Beer physicochemical parameters

Basic physicochemical parameters of the beers, such as alcohol content, extract content of the wort, real extract of the beer, degree of attenuation colour and energy content were measured using Anton Paar DMA 4500 densimeter and alcolyzer (Graz, Austria). Beer pH was analysed with the use Mettler Toledo MP 240 pH-meter (Columbus, DC, USA). Beer samples, prior to the analyses were degassed, mixed with diatomaceous earth (1 g per 100 cm^3^ of beer) and filtered through the laboratory filter paper. Six analyses were performed for each of the beers (two repetitions from three different bottles of the same beer).

#### Volatile compounds

Volatile compounds of the tested beers were analysed by the gas chromatography technique coupled with flame ionizing detection (GC–FID), using a GC2010 Plus apparatus with a FID-2010 and a headspace autosampler (HS-20) (Shimadzu Corporation, Kyoto, Japan), equipped with a CP-WAX 57 CB column (50 m × 0.32 mm ID × 0.2 µm) (Agilent Technologies, Santa Clara, CA, USA). Beer samples were degassed, mixed with diatomaceous earth (1 g per 100 cm^3^ of beer), and filtered through a paper filter. After filtration, 10 cm^3^ of beer was transferred to a 20 cm^3^ headspace vial. Each vial was conditioned in a headspace autosampler oven set at 40 °C and equilibrated for 20 min at shaking level 2 prior to the injection of the sample into the column. The operating conditions of the chromatographic analysis are described in detail in the work published by Kawa-Rygielska et al.^8^ Six measurements were performed for each of the beers (two repetitions from three different bottles of the same beer).

#### Identification and quantification of anthocyanins and phenolic acids

Anthocyanins and polyphenols in the tested beers were determined according to Kucharska et al.^9^ method using a Dionex HPLC (Walthman, MA, USA) system equipped with an Ultimate 3000 model of a diode array detector, an LPG-3400A quaternary pump, an EWPS-3000SI auto sampler, and a TCC-3000SD thermostated column compartment, controlled by Chromeleon v.7.2 software. The Cadenza Imtakt column CD–C18 (75 × 4.6 mm, 5 µm) was used. Anthocyanins in beers were detected at wavelength equal to 520 nm and phenolic acids at wavelength of 320 nm. Beers were degassed, centrifuged (6000 rpm, 10 min) and filtered through 0.45 µm PTFE syringe filters. The anthocyanins were expressed as peonidine 3-*O*-rutinoside (Peo3R) equivalents for peonidin 3-*O*-rutinoside-5-glucoside and peonidin 3-(*p*-coumaroyl)-rutinoside-5-glucoside; as petunidine 3-*O*-gluconiside (Pet3G) equivalents for petunidin 3-*O*-rutinoside-5-glucoside; as peonidine 3-*O*-gluconiside (Peo3G) equivalents for petunidin 3-(*p*-coumaroyl)-rutinoside-5-glucoside. The phenolic acids were expresses ad chlorogenic acid equivalent. Six analyses were performed for each of the beers (two repetitions from three different bottles of the same beer).

#### Carbohydrate profile and concentration of glycerol

The concentration of carbohydrates and glycerol were examined by the means of high-performance liquid chromatography (HPLC). Beer samples were degassed, centrifuged (6000 rpm, 10 min), diluted (1:1) with ultrapure water and filtered through syringe nylon filters (0.45 µm pore size) to chromatographic vials. The samples were then analyzed using a Prominence liquid chromatography system (Shimadzu Corp., Kyoto, Japan) equipped with a Rezex ROA-Organic Acid H + column (300 × 4.6 mm) from Phenomex (Torrance, CA, USA). The parameters of the measurements were previously described in the work of Pietrzak et al.^10^ Six analyses were performed for each of the beers (two repetitions from three different bottles of the same beer).

#### Concentration of phenolic compounds

Concentration of phenolic compounds of the beers was assessed using the Folin-Ciocalteu spectrophotometric method customized for beer, previously described by Kawa-Rygielska et al.^11^ Data were expressed as mg of gallic acid equivalent per dm^3^ of beer (GAE/dm^3^). Calibration curve (R value = 0.998) in the range of 0.30–9.00 mg GAE/cm^3^ was used to read the results. Nine analyses were performed for each of the beers (three repetitions from three different bottles of the same beer).

#### Free radical scavenging ability (ABTS^·+^ assay)

The antiradical ability of the beers was determined using ABTS^∙+^ assay customized for beer, described previously in the work by Kawa-Rygielska et al.^11^ The data were expressed as Trolox equivalent (TE) of antioxidative capacity per dm^3^ of the beer (mmol TE/dm^3^). Calibration curves, in the range 1.7–21.7 µmol TE/cm^3^, showing good linearity (r^2^ ≥ 0.999) were prepared using Trolox solution (0.005 mmol/dm^3^). Nine analyses were performed for each of the beers (three repetitions from three different bottles of the same beer).

#### Free-radical scavenging ability (DPPH· assay)

The antiradical ability of the beers was determined using DPPH∙ assay customized for beer, described previously in the work by Kawa-Rygielska et al.^11^ The data were expressed as Trolox equivalent (TE) of antioxidative capacity per dm^3^ of the beer (mmol TE/dm^3^). Calibration curves, in the range 2–10 µmol TE/cm^3^, showing good linearity (r^2^ ≥ 0.998) were prepared using Trolox solution (0.005 mmol/dm^3^). Nine analyses were performed for each of the beers (three repetitions from three different bottles of the same beer).

#### Ferric reducing (antioxidant) power (FRAP assay)

The antioxidant power of the beers was determined using FRAP assay customized for beer, described previously in the work by Kawa-Rygielska et al.^11^ The data were expressed as Trolox equivalent (TE) of antioxidant power per dm^3^ of the beer (mmol TE/dm^3^). Calibration curves, in the range 1.25–12.5 µmol TE/cm^3^, showing good linearity (r^2^ ≥ 0.998) were prepared using Trolox solution (0.005 mmol/dm^3^). Nine analyses were performed for each of the beers (three repetitions from three different bottles of the same beer).

#### Sensory analysis

Sensory analysis was performed by 10 trained panellists (6 men, 4 women, aged 27–40 years old). Beers were assessed by 7-point hedonic scale (1—strongly disliked, 2—moderately disliked, 3—slightly disliked, 4—neither liked nor disliked, 5—slightly liked, 6—moderately liked, 7—strongly liked) in parameters such as colour, clarity, taste, aroma and foaminess. Temperature of the served beer was 8 °C and beer was served in plastic clear cups. The samples were coded with random three digit numbers. Panellists were not acquainted with the details of the tested samples.

### Statistics

Data were processed using Statistica 13.5 software (Statsoft, Tulsa, OK, USA) using one way ANOVA (α = 0.05). Duncan test was used to analyse differences between means (*p* < 0.05) and to establish homogenous groups.

## Results and discussion

### Beer physicochemical parameters

Beers brewed with the use of purple potatoes were characterised with slightly different basic physicochemical parameters, shown in the Table 1.

**Table 1:** Physicochemical parameters of beers produced with the use of purple potatoes.

Parameter	C	P30	P50
Alcohol content [% v/v]	5.43 ± 0.06^a^	5.12 ± 0.09^b^	4.69 ± 0.08^c^
pH of the beer	4.11 ± 0.02^a^	4.18 ± 0.03^b^	4.25 ± 0.03^c^
Colour of the beer [EBC]	12.01 ± 0.14^c^	14.43 ± 0.26^b^	17.01 ± 0.31^a^
Extract of the wort [% w/w]	12.89 ± 0.11^a^	12.39 ± 0.13^b^	11.97 ± 0.08^c^
Real extract of the beer [% w/w]	4.70 ± 0.07^b^	4.86 ± 0.13^b^	5.26 ± 0.09^a^
Degree of attenuation [%]	78.66 ± 0.53^a^	73.43 ± 0.61^b^	71.31 ± 0.68^c^
Energy content [kcal]	46.57 ± 0.12^a^	44.77 ± 0.26^b^	43.11 ± 0.28^c^

^1^Values are expressed as a mean (n = 6) ± standard deviation. Mean values with different letters (a, b, c) within the same row are statistically different (α = 0.05) according to Duncan test.

Extract of the wort was lower for beers P30 and P50, which would be in accordance with the, for example lower glycerol content as well as lower amylase activity mentioned already in the Section “Concentration of anthocyanins and phenolic acids”. Attenuation of the beer, beer alcohol content as well as real extract of the beer further confirm the fact, that during the mashing more of the unfermentable sugars were produced in the beers P30 and P50^2^. Lower energy content is a consequence of the lower alcohol content of the beers brewed with the use of purple potato, as the ethanol is the main source of energy in beer and most of the alcoholic beverages.^12,13^ Lower pH of the beer C can also be easily explained by the lower alcohol content of the finished beer, as the beer pH decreases during the fermentation^2^. Typically beers with higher wort extract content ought to be darker (and acquire higher EBC parameter) due to the extraction of Maillard reaction type compounds from the malt^2^, but samples P30 and P50 were characterised with higher EBC parameter than the C. The concentration of anthocyanins is probably the main factor of the EBC parameter increase, as the sample C, characterised with the lowest EBC value possessed none of the anthocyanins and the EBC is the highest for P50, in which content of anthocyanins is the highest. The study published by Moirangathen et al.^14^ about the beer brewed with the use of black rice, in which they assessed the content of anthocyanins, acquired the same results: beers with highest anthocyanin content had highest EBC value.

### Volatile compounds

GC-FID analysis allowed for identification and quantification of 21 volatile components (8 alcohols, 4 aldehydes, 5 esters and 4 pyrazines) out of 30 analysed, which are shown in Table 2.

**Table 2:** Concentration of volatiles in the beers produced with the use of purple potatoes.

Compound	C	P30	P50
	mg/dm^3^		
Acetaldehyde	4.818 ± 0.664^a^	2.203 ± 0.044^b^	1.237 ± 0.009^c^
Propanal	0.701 ± 0.173^a^	0.604 ± 0.042^b^	0.621 ± 0.035^b^
Ethyl acetate	43.829 ± 1.252^a^	35.732 ± 3.786^b^	26.999 ± 1.198^c^
2-butanol	0.362 ± 0.001^b^	0.363 ± 0.001^b^	0.416 ± 0.003^a^
1-propanol	30.437 ± 3.244^c^	35.552 ± 1.185^b^	44.592 ± 7.102^a^
Hexanal	0.041 ± 0.004^a^	0.039 ± 0.002^a^	0.038 ± 0.002^a^
Isobutanol	63.787 ± 5.131^a^	44.232 ± 5.299^b^	43.852 ± 6.053^b^
Isopentyl acetate	2.607 ± 0.045^a^	1.572 ± 0.389^b^	0.978 ± 0.058^b^
1-butanol	0.119 ± 0.013^b^	0.225 ± 0.012^a^	0.250 ± 0.058^a^
2-methylbutanol	31.133 ± 2.531^a^	19.154 ± 2.728^b^	12.720 ± 3.739^c^
3-methylbutanol	77.399 ± 6.534^a^	51.420 ± 8.513^b^	36.040 ± 5.401^c^
Ethyl hexanoate	0.789 ± 0.004^a^	0.552 ± 0.023^b^	0.278 ± 0.027^c^
1-pentanol	4.627 ± 0.194^a^	0.747 ± 0.433^c^	1.741 ± 0.449^b^
2-methylpyrazine	0.338 ± 0.140^a^	0.282 ± 0.138^b^	0.031 ± 0.026^c^
2,5-dimethylpyrazine	0.347 ± 0.069^b^	0.409 ± 0.153^a^	0.317 ± 0.010^c^
2,3,5-trimethylpyrazine	2.044 ± 0.375^b^	2.644 ± 0.821^a^	0.812 ± 0.638^c^
Ethyl octanoate	1.505 ± 0.018^a^	1.335 ± 0.034^b^	0.962 ± 0.055^c^
Furfural	5.661 ± 1.061^a^	3.303 ± 0.071^b^	2.776 ± 0.030^c^
2,3-diethylpyrazine	0.391 ± 0.009^a^	0.385 ± 0.019^b^	0.375 ± 0.022^c^
Ethyl decanoate	3.252 ± 0.120^b^	4.288 ± 0.411^a^	3.349 ± 0.513^b^
Phenylethyl alcohol	46.551 ± 9.031^a^	17.549 ± 0.924^b^	4.450 ± 1.750^c^

^1^Values are expressed as a mean (n = 6) ± standard deviation. Mean values with different letters (a, b, c) within the same row are statistically different (α = 0.05) according to Duncan test.

Compounds such as izobutyraldehyde, butanal, 3-methylbutanal, methanol, 2,3-butanodione, ethyl heptanoate and 1-hexanol were not detected in the analysed beers. Beers brewed with the addition of potato were characterised with far lower total concentration of volatiles (182.39 mg/dm^3^ in P50 and 222.59 mg/dm^3^ in P30) than C (320.74 mg/dm^3^), albeit higher concentration of volatile components does not have to be an advantage, as many volatiles in the alcoholic beverages possess undesirable aroma, when they are present in the beer in great quantity^15^. Many of the so-called ‘higher alcohols’ or ‘fusel alcohols’, such as 1-propanol, 2-methylbutanol, 3-methylbutanol, 1-pentanol, phenylethyl alcohol and isobutanol belong to this category^16^. In the analysed beers an interesting influence of substituting barley malt with potatoes on the concentration of fusel alcohols can be seen. Higher percentage of potatoes in the ‘grain bill’ of the brewed beer resulted in lower concentration of 2-methylbutanol, 3-methylbutanol and phenylethyl alcohol, but concentration of 1-propanol and 1-butanol increased. Decrease in the concentration of 2-methylbutanol and 3-methylbutanol, as well as phenylethyl alcohol can be a result of far lower concentration of amino acids in the wort because *Saccharomyces cerevisiae* yeast produces these alcohols by the Ehrlich pathway, from the leucine, isoleucine and phenylalanine^16^. As the potatoes (typical potatoes and potatoes with coloured-flesh alike) possess lower concentration of these amino acids than barley, this explanation seems most plausible^17,18^. Different result can be seen in the concentration of 1-propanol. Samples P30 and P50 are characterised with higher concentration of this alcohol than the sample C. This alcohol is produced by the *Saccharomyces cerevisiae* yeast from amino acid threonine^19^. As the potatoes are not far richer in threonine than barley, then it can be assumed that during conditions of mashing, during which not all the proteins present in the malt are hydrolysed^2^, more threonine from the potatoes was released to the wort than from the barley malt. It is also interesting to note, that beers brewed with the addition of potato tubers were not characterised with methanol content. Typically, methanol is present in small amounts in various alcoholic beverages produced from the fruit, as the fruits can be characterised with the presence of small quantity of this alcohol and, more importantly, are source of pectins^20^. Pectins can be dagreded by pectinases produced by the *Saccharomyces cerevisiae* yeast, resulting in formation of minuscule amount of ethanol^20^. This is why, typically, beer posseses virtually none of methanol, in comparison to the beverages created from fruit musts, such as wines or wine distillates, as the barley malt is not a good source of pectins. On the other hand, potato tubers, in contrast to barley, are a rich source of various pectins, which should suggest, that beers brewed with the addition of potatoes should be characterised with methanol content^21^. However, beer-making process uses prolonged time of heat treatment during the mashing and wort boiling procedures and pectins are degraded by various thermal treatments, which means that in the resulting wort the concentration of pectins was too low to produce significant amounts of methanol^2,22^. The influence of addition of purple potato on two more compounds, very important in the beer brewing industry, should be closely analysed. Acetaldehyde is an intermediate in the production of many volatile compounds during the process of yeast fermentation, such as acetic acid (during the generation of acetyl-CoA and acetate esters) as well as ethanol, which is why the presence of this compound is rather unavoidable during the beer production. In low concentrations, this compounds is related to the pleasant green apple aroma, but higher content significantly worsens beverage flavour^23^. All the produced beers were characterised with the concentration of acetaldehyde in which it is not unpleasant and irritating^24^. However, it is worth to mention that the influence of potato addition to the produced beer resulted in far lower concentration of acetaldehyde than in control sample. P30 contained 45.72% of the acetaldehyde detected in C, while P50 contained just 25.67% of this volatile. This result should be seen as the advantage of using purple potatoes in the beer production technology, as acetaldehyde is far more toxic than the ethanol present in alcoholic beverages such as beer and wine^25,26^. Lower concentration of ethyl acetate, compound produced by yeast from glucose, might be the result of lower concentration of fermentable sugars in the worts from which the beers P30 and P50 were produced. High pressure liquid chromatography of beers analysed in this study confirmed, that beers P30 and P50 possess higher concentration of dextrins (which are unfermentable by *Saccharomyces cerevisiae*) and lower concentration of maltose and glucose^2^.

### Concentration of anthocyanins and phenolic acids

Beers produced with the use of purple potatoes were characterised with concentration of various anthocyanins and phenolic acids (shown in Table 3), which were absent in the control sample, which shows that only potatoes were source of these compounds.

**Table 3:** Concentration of anthocyanins and phenolic acids in beers produced with the use of violet potatoes.

Compound	C^1^	P30	P50
	mg/dm^3^		
Petunidin 3-*O*-rutinoside-5-glucoside	n.d	1.059 ± 0.257^b^	2.771 ± 0.84^a^
Peonidin 3-*O*-rutinoside-5-glucoside	n.d	1.580 ± 0.752^b^	5.686 ± 1.683^a^
Petunidin 3-(*p*-coumaroyl)-rutinoside-5-glucoside	n.d	7.310 ± 0.738^b^	19.868 ± 4.551^a^
Peonidin 3-(*p*-coumaroyl)-rutinoside-5-glucoside	n.d	2.434 ± 0.252^b^	8.182 ± 1.962^a^
Chlorogenic acid	n.d	115.815 ± 4.911^b^	237.147 ± 14.263^a^
Cryptochlorogenic acid	n.d	120.433 ± 11.832^b^	250.684 ± 15.684^a^
Neochlorogenic acid	n.d	124.259 ± 20.195^b^	233.724 ± 14.561^a^

^1^Values are expressed as a mean (n = 6) ± standard deviation. Mean values with different letters (a, b) within the same row are statistically different (α = 0.05) according to Duncan test. n.d.—compound not detected.

Four anthocyanins and three phenolic acids were identified and quantified in the samples P30 and P50. Total concentration of anthocyanins in P30 was equal to 12.383 mg per dm^3^ and in P50 equal to 36.507 mg per dm^3^ of beer. The most abundant anthocyanin in both beer samples was petunidin 3-(*p*-coumaroyl)-rutinoside-5-glucoside and its concentration exceeded 50% of total anthocyanin content. Concentration of these substances in potatoes of purple-coloured flesh is usually far higher, ranging for substances such as petunidin 3-(*p*-coumaroyl)-rutinoside-5-glucoside from 22.4 to 86.2 mg per 1 kg of potatoes (fresh weight)^27^. However, it is not surprising that not all the anthocyanins are transferred to the wort via mashing, as the main goal of this process is not the extraction of anthocyanins and polyphenols, but hydrolysis of starch and proteins^2^. Additionally, more of the anthocyanins can perish during the long boiling process of the wort, because heat-treatment is one of the most important factors which degrade anthocyanins^28^. Study conducted by Tierno et al. about boiling the potato tubers have shown, that 30 min boiling process can reduce anthocyanin content of anthocyanins in purple-flesh coloured potatoes from 17.6 to 72.8%^29^. Furthermore, *Saccharomyces cerevisiae* yeast is capable of anthocyanin adsorption as well as biotransformation which might further decrease the concentration of anthocyanins in beverage^30,31^. Morata et al. in a 2005 study about anthocyanin adsorption of anthocyanins determined, that various *Saccharomyces cerevisiae* yeast are able to adsorb as much as 30.91 mg of anthocyanins from a litre of fermented beverage (with average of 18.57 mg adsorbed), which might further explain rather low concentration of anthocyanins in the beer brewed with the addition of purple potato^32^. Beers brewed with the addition of purple potato are characterised with far greater concentration of phenolic acids than anthocyanins. Three phenolic acids, such as chlorogenic acid, cryptochlorogenic acid and neochlorogenic acid were identified in P30 and P50. Concentration of these compounds was far greater than concentration of anthocyanins and was equal to 360.507 mg for P30 and 721.555 mg for P50. Such great difference between the concentration of phenolic acids and anthocyanins in the beer ought not to be surprising, as the coloured potatoes possess significantly higher concentration of chlorogenic, neochlorogenic and cryptochlorogenic acid than anthocyanins^33,34^. Additionally, these three acids are characterised with high stability during the heat treatment and in the low pH environment which easily explains higher concentrations of these particular chemicals in the prepared beverages^35,36^.

### Concentration of carbohydrates and glycerol

Table 4 shows concentration of carbohydrates (glucose, maltose, maltotriose and dextrinsand glycerol in beers brewed with the use of potatoes with purple-coloured flesh.

**Table 4:** Concentration of carbohydrates and glycerol in the beers produced with the use of purple potatoes.

Compound	C^1^	P30	P50
	mg/dm^3^		
Glucose	0.312 ± 0.016^a^	n.d	n.d
Maltose	0.775 ± 0.121^a^	0.264 ± 0.062^b^	0.178 ± 0.011^c^
Maltotriose	2.144 ± 0.363^a^	0.693 ± 0.143^b^	0.264 ± 0.068^c^
Dextrins	34.323 ± 1.522^c^	35.589 ± 1.172^b^	37.853 ± 2.086^a^
Glycerol	2.604 ± 0.512^a^	2.324 ± 0.397^ab^	2.179 ± 0.338^b^

^1^Values are expressed as a mean (n = 6) ± standard deviation. Mean values with different letters (a, b) within the same row are statistically different (α = 0.05) according to Duncan test. n.d.—compound not detected.

Beers P30 and P50 were characterised with higher concentration of unfermentable dextrins, with P50 possessing more dextrins than P30, which suggests, that the starch present in potatoes hydrolyses into less fermentable sugars than barley starch, albeit it might be also a result of lack of proper concentration of beta-amylase enzyme, which cleaves maltose from the starch. With lower amount of barley malt in the ‘grain bill’ of the produced beer, concentration of amylases in the mash decreased, which might result in inadequate activity of amylases during the process of beer production. Therefore, in the beers P30 and P50 more time would be needed during mashing at the temperature of 62 °C (which is optimal for beta-amylase activity) to produce as much fermentable sugars as in the sample C^2^. Additionally, lower concentration of fermentable sugars in the worts used to produce beers P30 and P50 would also explain lower concentration of glycerol in the analysed samples, because glycerol is produced by the *Saccharomyces cerevisiae* yeast as the by-product of ethanol fermentation^37,38^.

### Concentration of phenolic compounds and antioxidant activity of the beers

The total phenolic content of the beer as well as beer antioxidant activity is shown in the Table 5.

**Table 5:** Total phenolic content (TPC) and antioxidant activity (ABTS, DPPH, FRAP) of the beers produced with the use of purple potatoes.

	C^1^	P30	P50
TPC [mg GAE^2^/1dm^3^]	244.0 ± 10.8^c^	261.5 ± 09.1^b^	300.5 ± 11.1^a^
ABTS^+^∙ assay [mmol TE^3^/1dm^3^]	1.88 ± 0.05^c^	2.01 ± 0.07^b^	2.38 ± 0.10^a^
DPPH∙ assay [mmol TE/1dm^3^]	0.60 ± 0.04^c^	0.86 ± 0.02^b^	1.26 ± 0.05^a^
FRAP assay [mmol TE/1dm^3^]	1.63 ± 0.05^c^	1.87 ± 0.04^b^	2.06 ± 0.05^a^

^1^Values are expressed as a mean (n = 9) ± standard deviation. Mean values with different letters (a, b) within the same row are statistically different (α = 0.05) according to Duncan test.

^2^GAE—gallic acid equivalent.

^3^TE—trolox equivalent.

The use of potatoes with purple-coloured flesh increased concentration of phenolic compounds in the beers, as well as the antioxidant activity of the beverages. As the main phenolic compounds in the beers originate from barley, which is not especially rich in phenolic compounds, changing part of the ‘grain bill’ of the beer for potatoes with purple-coloured flesh, which have been proven to possess high concentration of phenolic compounds, as well as high antioxidant activity (Rytel et al.; Rytel et al.; Lachman et al.^34^) ought to increase phenolic compound content, as well as increase beer antioxidant activity, which is confirmed by the results acquired in this study^2,31,39–41^. The increased concentration of chlorogenic acid, neochlorogenic acid and cryptochlorogenic acid, as well as anthocyanins is probably the main reason of the increased activity of the P30 and P50 beers, because these compounds have been proven in the past to be strong antioxidants^42,43^.

### Sensory analysis

The results of the sensory analysis (Table 6) have shown that P50 is inferior to the P30 and C beer in most of the assessed parameters.

**Table 6:** Sensory analysis of the beers produced with the use of purple potatoes.

Parameter	C^1^	P30	P50
Colour	6.80 ± 0.13^a^	6.60 ± 0.16^ab^	6.20 ± 0.25^b^
Clarity	6.40 ± 0.16^a^	6.20 ± 0.43^a^	2.60 ± 0.26^b^
Taste	5.80 ± 0.39^a^	5.20 ± 0.44^ab^	4.60 ± 0.34^b^
Aroma	5.00 ± 0.47^a^	5.20 ± 0.44^a^	3.20 ± 0.13^b^
Foaminess	5.40 ± 0.45^a^	5.20 ± 0.33^a^	2.60 ± 0.34^b^

^1^Values are expressed as a mean (n = 10) ± standard deviation. Mean values with different letters (a, b) within the same row are statistically different (α = 0.05) according to Duncan test.

Nevertheless, P50 still acquired high score (6.20) for the colour parameters. P30 and C were similarly rated in all of the tested parameters, with P30 acquiring slightly lower score in the taste parameter. Panellists described beer P50 as one, which had ‘foreign’ taste and smell and ‘unpleasant’ aftertaste. These results show that 30% of extract in the brewed beer is the appropriate dosage of potatoes which can be easily used without need of any further changes in the beer recipe to acquire palatable beverage. Still, it is important to mention, that addition of unusual substrates into the beer production often results in changing sensory attributes of the beer, however, by applying appropriate changes into the beer production regime, most of the disadvantages can be mitigated^2^. For example, studies of Vrzal et al. (2021) have shown that changes in the amino acids content in the finished beer correspond with changes in sensory properties of the beer in the aspects of beer ‘fullness’, ‘astringency’, ‘sourness’ or ‘sweetness’, therefore by applying changes to the protein rest during the mashing procedure, beer brewers could influence aroma of the beer brewed with addition of potatoes^44^. Similarly, various malt additives, such as dark malts or caramel malts could be used during the beer brewing to change taste and aroma of the produced beverage^45^. Additional method, which is gaining popularity nowadays, is using unconventional yeast or other microorganisms (such as lactic acid bacteria) during the process of beer fermentation to modify taste, aroma, attenuation degree or even colour of the produced beer^46^. Beer taste, presence and aroma can be also modified in great extent by addition of various hops and fruits^46,47^.

## Conclusions

Results acquired in this study show, that there is a viable possibility of using potatoes with purple-coloured flesh in the beer brewing technology. Produced beers acquired rather satisfactory physicochemical properties important in the beer production. Additionally, brewing beer with the addition of potatoes with purple-coloured flesh resulted in beers with higher concentration of polyphenolic compounds, higher antioxidant activity and lower calorie value and alcohol content. Analysis of volatile components in the beers brewed with the use of potatoes with purple-coloured flesh showed that these beers possess the whole range of compounds characteristic for pilsner-type beers, but usually in lower quantities than in control beer. Further studies on the beers brewed with addition of potatoes could be concentrated on the adjustment of the beer brewing conditions to combine novel pro-health benefits of compounds with acceptable organoleptic quality of finished product.

## Data Availability

The datasets used and analysed during the current study are available from the corresponding author on reasonable request.
